# A 2-Gene Signature Related to Interferon-Gamma Predicts Prognosis and Responsiveness to Immune Checkpoint Blockade of Glioma

**DOI:** 10.3389/fmed.2022.846847

**Published:** 2022-04-15

**Authors:** Yongzhe Li, Hang Ji, Xin Gao

**Affiliations:** ^1^Department of Neurosurgery, Second Affiliated Hospital of Harbin Medical University, Harbin, China; ^2^Translational Medicine Research and Cooperation Center of Northern China, Heilongjiang Academy of Medical Sciences, Harbin, China

**Keywords:** glioma, IFNGR1, IFNGR2, prognostic, tumor relapse, immune checkpoint blockade therapy

## Abstract

**Background:**

Gliomas represent the most common and aggressive brain malignancy. Interferon-gamma (IFNG) is a potent inducer of immune response, developing IFNG-related gene signature may promote the diagnosis and treatment of this disease.

**Methods:**

Bulk tumor and single-cell mRNA-seq datasets of glioma ranging from WHO grade II to IV with corresponding demographics were included. Multiple bioinformatics and machine learning algorithms were performed to develop an IFNG-related prognostic signature and evaluate immune checkpoint blockade (ICB) therapy response.

**Results:**

IFNGR1 and IFNGR2 were used as concise IFNG-related gene signature based on which the IFNGR score well-characterized the IFNG response in the glioma microenvironment. Increased IFNGR score was associated with clinicopathological parameters relating to tumor malignancy and prevailing molecular pathological markers. Notably, K-M and Cox regression analysis found that the IFNGR score was an effective prognostic biomarker, and was associated with tumor relapse for a subset of patients. Notably, IFNGR1 and IFNGR2 were preferentially expressed by the Mono/Macro cells in the glioma microenvironment and were significantly correlated with M2 macrophage. Thus, the IFNGR score-high group had increased expression of immune checkpoints and had the potential to predict ICB responsiveness.

**Conclusion:**

In conclusion, we have developed a concise IFNG-related gene signature of clinical significance, which may improve the current diagnosis and treatment of glioma.

## Introduction

Glioma is the most common and lethal primary brain malignancy worldwide, accounting for 31.1% of primary brain tumors in people aged 20–59 years (1, 2). The overall survival (OS) of gliomas varies widely, from 78.1 months for low-grade glioma (WHO grade II) to 14.4 months for glioblastoma (GBM) (WHO grade IV), with 5-year survival rates ranging from 67% for low-grade glioma to 9% for GBM. Despite currently standard treatment, malignant progression and tumor relapse inevitably occur and lead to tolerance to conventional treatments (1–3). Therefore, novel biomarkers are in urgent need to assist in the accurate diagnosis and improve the currently limited treatment efficacy.

Accumulative evidence has demonstrated the paramount role of the IFNG response in tumors (4, 5). On the one hand, IFNG is a potent inducer of the adaptive immune response, promoting antigen presentation and effector T cell activity, and catalyzing immune-mediated tumor clearance (6, 7). On the other hand, IFNG participants in immune editing and upregulates immune checkpoints to facilitate tumor cells evade immune attacks (7, 8). Notably, substantial evidence has suggested that gene signatures associated with the IFNG response reliably predict prognosis and responsiveness to ICB of tumor sufferers (4, 9), while developing IFNG-related gene signature for both GBM and lower-grade glioma (LGG) was less addressed. As the portal of the IFNG signaling pathway, IFNGR1 and IFNGR2 are of biological and pathophysiological significance. Biologically active IFNGR-IFNG complex needs two IFNGR1 and two IFNGR2, with one of each receptor subunit binding to each end of the IFNG homolog, thereby activating the downstream JAK and STAT families and enabling the biological effects of IFNG (7, 10). The dysregulation of IFNGR1 and IFNGR2 results in immune-related disorders (11–14). Therefore, IFNGR1 and IFNGR2 may also constitute a genetic metric of clinical implications in glioma.

From this perspective, we integrated IFNGR1 and IFNGR2 as an IFNG related 2-gene signature and developed the IFNGR score to probe their clinical significance. We thoroughly explored the association between the IFNGR score and glioma clinicopathological and molecular features, as well as the underlining molecular mechanism. Notably, the IFNGR score effectively predicted the OS and progression-free interval (PFI) of glioma and may act as a potential predictor of ICB therapy. On this basis, our results provide an efficient classifier for determining glioma prognosis and may contribute to optimizing conventional treatment of the desperate disease.

## Materials and Methods

### Data Collection and Pre-procession

mRNA-seq datasets of a total of 1,693 glioma samples from WHO grade II to grade IV and corresponding demographics were retrieved (Table 1). Of these, 675 glioma samples of the Cancer Genomic Atlas (TCGA) project were retrieved from the USCS Xena data portal (https://xenabrowser.net/datapages/) and were defined as the exploration dataset. The remaining samples were from the Chinese Glioma Genome Atlas (CGGA, http://www.cgga.org.cn/index.jsp) were defined as the validation datasets (CGGA693, *n* = 693; CGGA325, *n* = 325) (15–18). Samples with no follow-up information or follow-up time of <1 day were excluded. Genes with 0 (not detected) expression in more than half of the samples were removed. For mRNA-seq data sets, the gene expression profile was TPM normalized and log-transformed for downstream analysis. The scRNA-seq dataset was retrieved from the TISCH database (http://tisch.comp-genomics.org/home/) (19). GSE 131928 10X dataset was included in our study (20).

**Table 1:** Patient clinical characteristics of the TCGA and CGGA cohorts.

**Features**	**TCGA**	**CGGA693**	**CGGA325**
**Age**			
>60	135 (20.00%)	71 (10.25%)	24 (7.38%)
< =60	482 (71.41%)	621 (89.61%)	301 (92.62%)
**Gender**			
Male	360 (53.33%)	398 (57.43%)	203 (62.46%)
Female	257 (38.07%)	295 (42.57%)	122 (37.54%)
**WHO grade**			
WHO grade II	216 (32.00%)	188 (27.13%)	103 (31.69%)
WHO grade III	241 (35.70%)	255 (36.80%)	79 (24.31%)
WHO grade IV	160 (23.70%)	249 (35.93%)	139 (42.77%)
**IDH status**			
Wildtype	237 (35.11%)	286 (41.27%)	149 (45.85%)
Mutant	429 (63.56%)	356 (51.37%)	175 (53.85%)
**MGMTp methylation**			
Methylated	478 (70.81%)	315 (45.45%)	157 (48.31%)
Unmethylated	163 (24.15%)	227 (32.76%)	149 (45.85%)

### Development of the Gene Signature and Functional Analysis

The IFNGR score was defined as the ssGSEA score of IFNGR1 and IFNGR2 based on the ssGSEA algorithm (21). Samples were then split into the IFNGR score-high and -low groups by the median value. To demonstrate the efficacy of the IFNGR score in characterizing the IFNG response in gliomas, we collected genesets previously developed associated with IFNG response, including IFNG.1 (GBP5, ICAM1, CAMK2D, IRF1, SOCS3, CD44, and CCL2) (4), IFNG.2 (IDO1, CXCL10, CXCL9, HLA-DRA, STAT1, and IFNG) (9), and IFNG.ex (CD3D, IDO1, CIITA, CD3E, CCL5, GZMK, CD2, HLA-DRA, CXCL13, IL2RG, NKG7, HLA-E, CXCR6, LAG3, TAGAP, CXCL10, STAT1, and GZMB) (9). Samples were split into the IFNG.1 score-high and -low groups, IFNG.2 score-high and -low groups, and IFNG.ex score-high and -low groups following the methods described before (4, 9). Gene set enrichment analysis (GSEA, v4.1.0) was conducted to calculate the normalized enrichment score (NES) of the IFNG signaling pathway, and biological processes relating to IFNG response (22). Differentially expressed genes (DEGs) between the IFNGR score-high and -low groups were calculated using the R packages “limma” and “edgeR.” Functional enrichment analysis was further employed to exhibit the function of DEGs based on the webtool DAVID (https://david.ncifcrf.gov/) (23–26). Tumor purity was estimated using the ABSOLUTE algorithm (27).

To examine the expression of IFNGR1 and IFNGR2 at the cellular level, scRNA-seq expression profile was analyzed using the R package “Seurat” (28). In brief, the sample was sequentially normalized, dimensionality reduced, and clustered. A total of 22 clusters were identified and cell type identification was based on differentially expressed genes defined by Neftel et al. and the CellMarker database (http://bio-bigdata.hrbmu.edu.cn/CellMarker/) (29). We defined subgroups expressing multiple cellular markers as “Unclassified.” Thereafter, the expression profile of the Mono/Macro subcluster was extracted and the ssGSEA score of IFNGR1 and IFNGR2 was calculated using the ssGSEA algorithm. DEGs between the score-high and -low groups were evaluated using “Seurat.” Transcription factor enrichment analysis was performed using the web tool Metascape (https://metascape.org/gp/index.html) based on the TRRUST dataset (30, 31). The fraction of 22 immune infiltrations was estimated by the CIBERSORT algorithm (32).

### Validation of Clinical Significance

The Kaplan-Meier (K-M) plots were used to exhibit the survival differences and univariate Cox regression analysis was conducted to assess the independent prognostic significance based on the R packages “survival” and “survminer.” The receiver operating curves (ROC) and corresponding area under the curve (AUC) were employed to evaluate the time-dependent predictive power. TIDE is a computational framework for modeling the induction of T cell dysfunction in tumors with high infiltration of cytotoxic T lymphocytes and the prevention of T cell infiltration in tumors with low cytotoxic T lymphocyte infiltration level (http://tide.dfci.harvard.edu/login/). We employed the webtool TIDE to predict the responsiveness of samples to ICB therapies with default parameters, and the predicted results were further corrected by a machine learning algorithm, SubMap, as the expression profile and corresponding response to ICI of melanoma being the reference (33–35).

### Statistics

All the statistics were performed in R (version 4.0.2). The IFNGR score between groups was compared using the two-tailed Wilcox test. K-M analysis and log-rank test were used to assess survival differences. ROC curves and corresponding AUCs were used to assess the time-dependent predictive power. Univariate Cox regression analysis was employed to describe the independent prognostic value. *p* < 0.05 was considered statistically significant. For GO and GSEA analysis, a false discovery rate (FDR)-q value < 0.1 was considered significant. For the predicted results of ICB responsiveness, Bonferroni corrected *p* < 0.1 was considered significant. We marked ^*^ for *p* < 0.05, ^**^ for *p* < 0.01, ^***^ for *p* < 0.001.

## Results

### IFNGR1 and IFNGR2 Make Up a Concise Gene Signature Characterizing IFNG Response

**Figure 1:**
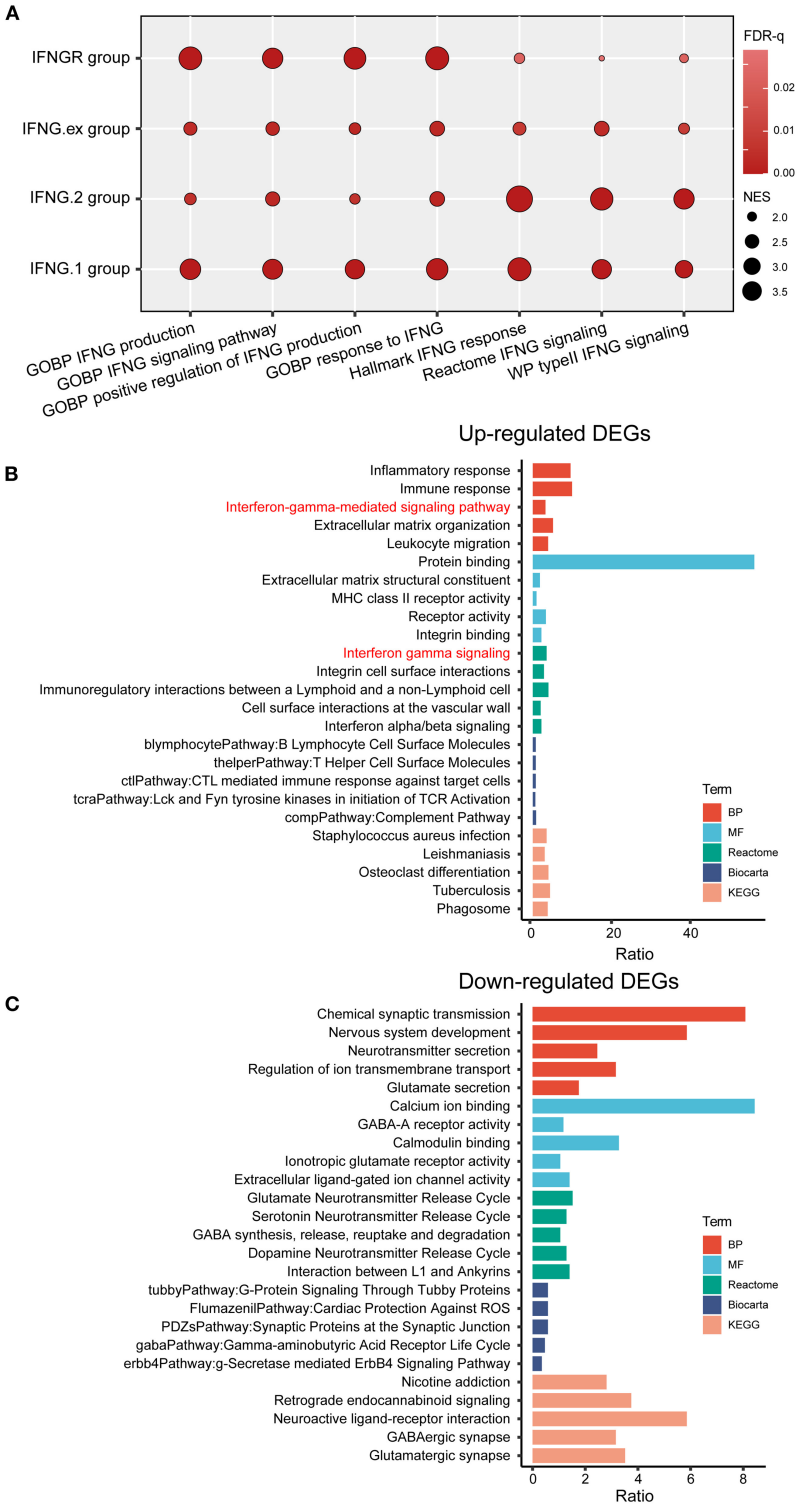
The IFNGR1 and IFNGR2-based gene signature in characterizing the IFNG response in gliomas. **(A)** GSEA analysis of the IFNGR, IFNG.1, IFNG.2, and IFNG.ex-based group in characterizing the IFNG signaling pathway and related biological processes. The size of the bubble was proportional to the NES score and color was proportional to the FDR-q value of each term. GO analysis of the **(B)** up-regulated and **(C)** down-regulated DEGs between the IFNGR score-high and -low groups based on the TCGA cohort. The top 5 terms in each category were exhibited.

We first explored the efficacy of IFNGR1 and IFNGR2 as a genetic metric to characterize the IFNG response. The IFNGR score was calculated based on the expression of IFNGR1 and IFNGR2 and samples were split into the IFNGR score-high and -low groups by the median value. Meanwhile, samples were split into the IFNG.ex score-high and -low groups, IFNG.1 score-high and -low groups, and IFNG.2 score-high and -low groups based on previously constructed IFNG-related gene signatures. The activity of the IFNG signaling pathway (Hallmark IFNG response, Reactome IFNG signaling, and WP type II IFNG signaling) and IFNG-related process (GOBP IFNG signaling, IFNG production, and positive regulation of IFNG production) in the two groups were compared. As a result, the IFNGR score-high group had the highest average NES scores in IFNG-related BP (mean NES = 3.26), while the lowest NES scores in the IFNG signaling pathway (mean NES = 1.93) (Figure 1A). Instead, IFNG.2-based group best characterized the IFNG signaling pathway well (mean NES = 3.44), while scored lower in IFNG-related biological processes (mean NES = 2.16), and the IFNG.1-based group was balanced in characterizing the IFNG signaling pathway (mean NES = 2.97) and related biological processes (mean NES = 3.00). Moreover, DEGs (IFNGR score-high vs. score-low) were calculated, and GO analysis found that interferon-gamma-mediated signaling pathway (BP) and interferon-gamma signaling (Reactome) were top enriched terms in the IFNGR score-high group (Figures 1B,C). Notably, inflammatory response, immune response, leukocyte migration (BP), and signaling pathways regulating the activity of immune cells including integrin cell surface interaction (Reactome), immunoregulatory interaction between a lymphoid and a non-lymphoid cell (Reactome), B lymphocyte, and T helper cell surface molecule (Biocarta), and CTL-mediate immune response against target cell (Biocarta) were also highly enriched terms of the IFNGR score-high group, indicating a pro-inflammatory microenvironment which corroborates the function of IFNG. Therefore, IFNGR1 and IFNGR2 comprised of a concise gene signature effectively characterize the IFNG response.

### The IFNGR Score Was Associated With the Function State of Glioma-Associated Macrophages

**Figure 2:**
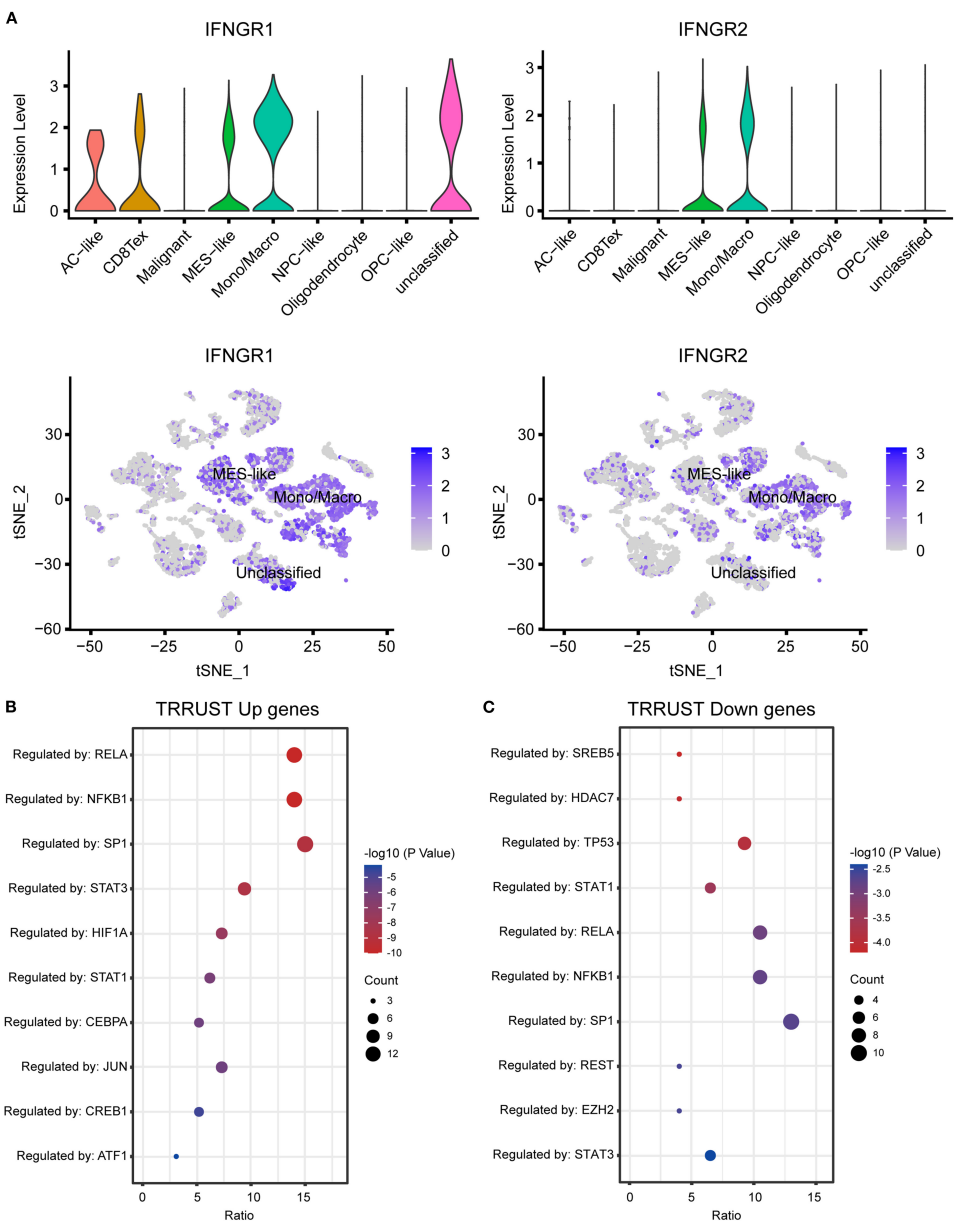
Association of IFNGR1 and IFNGR2 expression with glioma-associated macrophages. **(A)** The expression of IFNGR1 and IFNGR2 in each cell type. Enrichment analysis of transcription factors between IFNGR1 and IFNGR2 ssGSEA score-high and -low groups, **(B)** upregulated DEGs (logFC > 0.5) were subscribed to the translational regulation of preferentially NF-κB and STAT3, and **(C)** downregulated DEGs were subscribed preferentially to STAT1.

Next, we explored the expression of IFNGR1 and IFNGR2 at single-cell resolution. Twenty two 22 clusters of cells were identified and 8 types of cells were annotated. A cluster of cells expressing a mixture of markers of malignant cell, MES-like malignant cell, and monocyte/macrophage was defined as unclassified and excluded from further analysis. IFNGR1 was expressed by Mono/Macro, unclassified cell, MES-like malignant cell, AC-like malignant cell, and CD8 T cell. Mono/Macro subcluster had the highest expression level (Figure 2A). On the other hand, Mono/Macro and MES-like malignant cells express IFNGR2 and the former had remarkably increased value. Moreover, we extracted the expression profile of the Mono/Macro subcluster and calculated the ssGSEA score of IFNGR1 and IFNGR2, and split the Mono/Macro cells into score-high and -low groups by the median value. DEGs were calculated and transcription factor enrichment analysis based on the TRRUST database found that NF-κB and STAT3 were preferentially activated transcription activators in the score-high group (Figure 2B), possibly indicating that increased IFNGR1 and IFNGR2 expression was associated with an alternative activation state of macrophages in the glioma microenvironment. In turn, STAT1 was activated at a higher level in the score-low group (Figure 2C), which may indicate a classical activation state of the macrophages.

### The IFNGR Score Was Indicative of a Malignant Phenotype of Glioma

**Figure 3:**
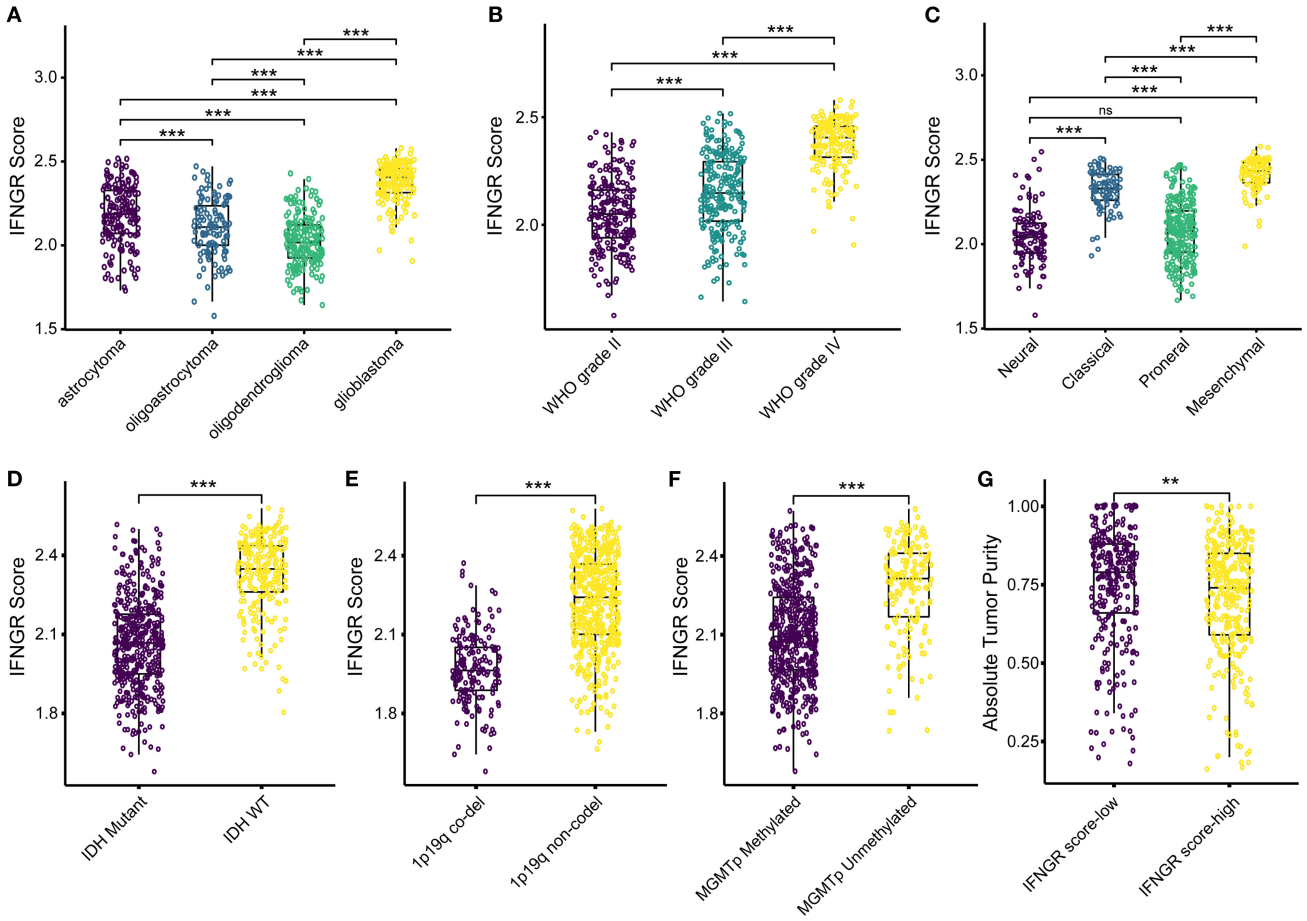
The association between the IFNGR score and clinicopathological parameters of glioma. The distribution of the IFNGR score in **(A)** histology class, **(B)** WHO grade, and **(C)** transcriptome subtype. The association between the IFNGR score and molecular pathology biomarkers, including **(D)** IDH mutation status, **(E)** 1p19q co-deletion status, and **(F)** MGMT promoter methylation status. **(G)** Tumor purity estimated using ABSOLUTE algorithm between the IFNGR score-high and -low groups. ** represents *p* < 0.01, *** represents *p* < 0.001.

Then we explored the association between the IFNGR score and prevalent clinicopathological features of glioma. In terms of histology, the IFNGR score was lowest in oligodendroglioma and sequentially increased in oligoastrocytoma, astrocytoma, and glioblastoma (Figure 3A). Likewise, the IFNGR score increased with the WHO tumor grade, with WHO grade IV having the highest IFNGR scores (Figure 3B). Among the transcriptome subtype of glioma, the IFNGR score was lower in the neural and proneural subtypes and highest in the mesenchymal subtype (Figure 3C). In terms of molecular pathology biomarkers, samples of IDH1 wildtype tended to have increased IFNGR scores (Figure 3D). Patients with 1p19q co-deletion had decreased IFNGR score, possibly because 1p19q co-deletion was associated with oligodendroglial histology of glioma (Figure 3E). Besides, samples with unmethylated MGMT promoters had higher IFNGR scores (Figure 3F). Moreover, samples with increased IFNGR scores had decreased tumor purity, in line with the putative pro-inflammatory microenvironment of the IFNGR score-high group (Figure 3G). Together, these results demonstrated that increased IFNGR scores were likely to indicate a high degree of malignancy in gliomas.

### An Increased IFNGR Score Was Indicative of Poor Prognosis and Short-Term Glioma Relapse

**Figure 4:**
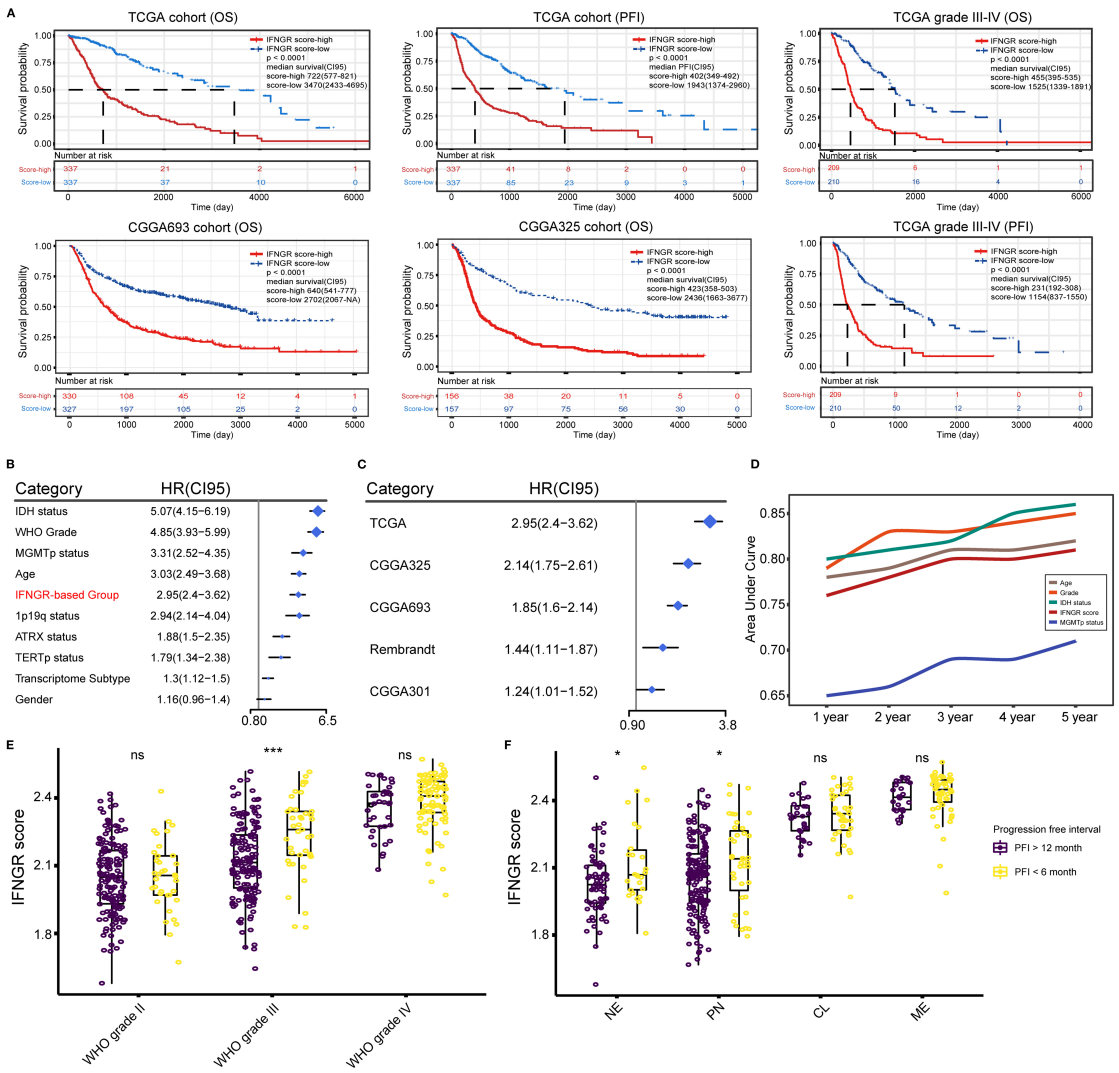
Prognostic significance of the IFNGR-based group. **(A)** K-M analysis of the IFNGR-based group in terms of OS and PFI based on TCGA, CGGA693, and CGGA325 cohorts. The prognostic value of IFNGR-based group in WHO grade III and IV gliomas was also exhibited. **(B)** Univariate Cox regression of the independent prognostic significance of the IFNGR-based group and other clinicopathological parameters based on the TCGA cohort. **(C)** Univariate Cox regression analysis of the independent prognostic significance of the IFNGR-based group based on TCGA, CGGA693, and CGGA325 group, as well as two external validation data sets (Rembrandt, *n* = 476, and CGGA301, *n* = 301). **(D)** Time-dependent ROC and corresponding AUCs of age, WHO tumor grade, IDH mutation status, IFNGR score, and MGMT promoter methylation status. The association between the IFNGR score and tumor early (PFI < 6 months) and delayed (PFI > 12 months) relapse in different tumor classifications including **(E)** WHO tumor grade, and **(F)** transcriptome subtype. * represents *p* < 0.05.

Next, we investigated the prognostic significance of the IFNGR score. In the TCGA cohort, an increased IFNGR score strongly suggested a poor prognosis (*p* < 0.0001, median survival of 722 days in the IFNGR score-high group), along with a significantly decreased progression-free interval (PFI) (*p* < 0.0001, median PFI of 402 days in the IFNGR score-high group) (Figure 4A). Similar results were yielded in the CGGA325 and CGGA693 cohorts (*p* < 0.0001, the median survival of 423 and 640 days in the IFNGR score-high group, respectively), as well as in the WHO grade III-IV gliomas. Besides, univariate Cox regression analysis showed that the IFNGR-based group had independent risk prognostic significance, with an HR of 2.95 (with the IFNGR score-low group being the reference), suggesting a nearly 3-fold increase in mortality of patients in the IFNGR score-high (Figure 4B). Notably, the IFNGR-based group held up as an independent risk prognostic factor in multiple datasets with HRs ranging from 2.95 (TCGA) to 1.24 (CGGA301) (Figure 4C). Moreover, time-dependent ROC analysis exhibited 1- to 5-year AUC values of 76, 78, 80, 80, and 81% for the IFNGR-based group, second to age, WHO grade, and IDH mutation-based groups, and superior to MGMT promoter (Figure 4D). Given the significantly improved PFI in the IFNGR score-low group, the association between the IFNGR score and tumor relapse was further explored. Samples were divided into short-term relapse group (PFI < 6 months, *n* = 177) and delayed relapse group (PFI > 12 months, *n* = 389). For patients who suffered from WHO grade III glioma, the IFNGR score was significantly higher in the early relapse group (*p* < 0.001) (Figure 4E). In terms of transcriptome subtypes, samples of NE and PN subtypes with increased IFNGR scores tended to have short-term tumor recurrence (*p* < 0.05) (Figure 4F).

### The IFNGR-Based Group Predicts the ICB Responsiveness of Glioma

**Figure 5:**
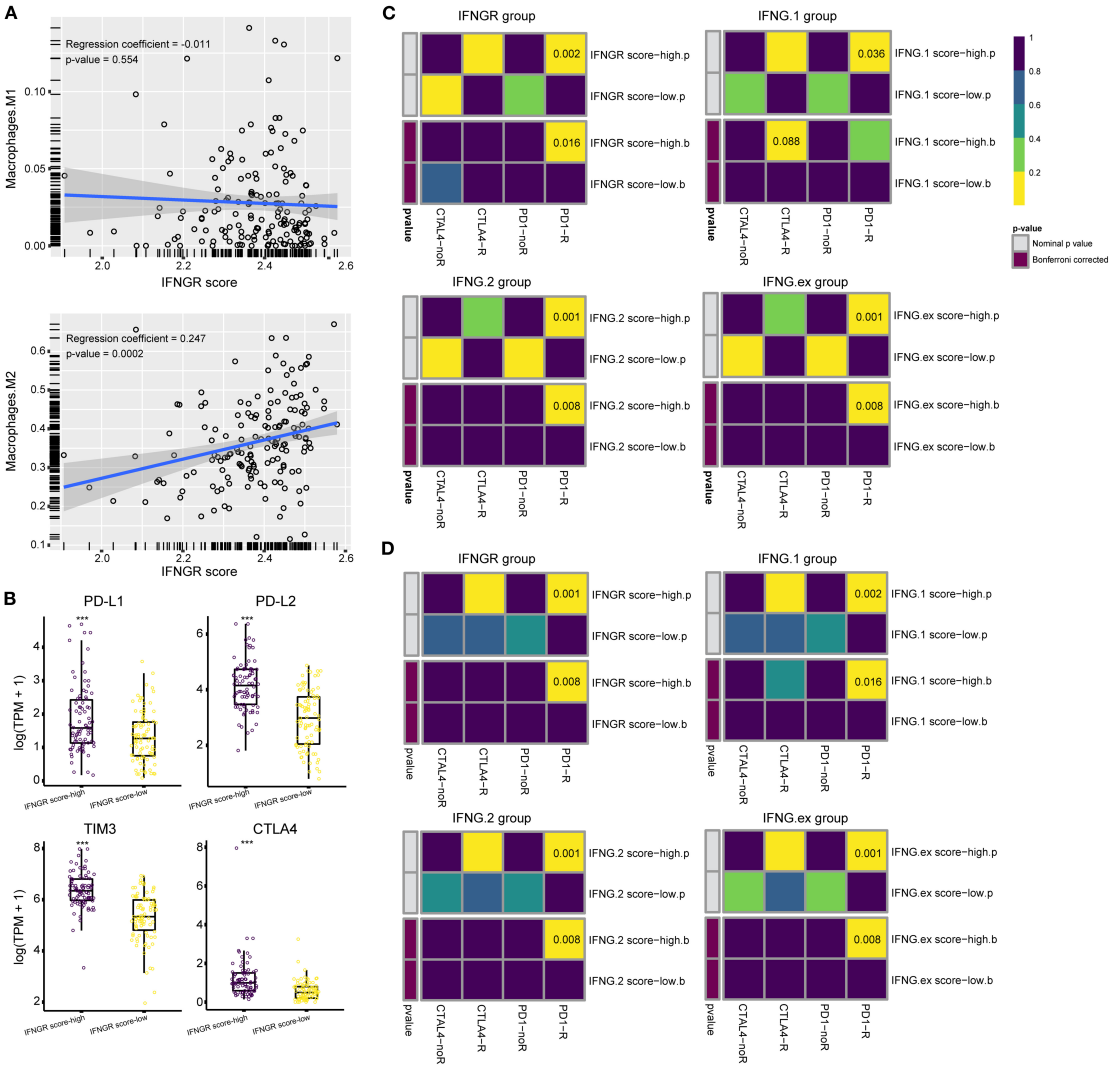
The IFNGR-based group predicts ICB responsiveness of glioma. **(A)** Correlation analysis between the IFNGR score and M2 fraction inferred by CIBERSORT. **(B)** The expression of immune checkpoints between the IFNGR score-high and -low groups. TIDE algorithm estimates the ICB responsiveness of the IFNGR score, IFNG.1, IFNG.2, and IFNG.ex-based group using the **(C)** TCGA and **(D)** CGGA325 cohort. The predicted results were further corrected by a machine learning algorithm, SubMap. *** represents *p* < 0.001.

Lastly, we interrogated whether the IFNGR score had the potential to predict the ICB responsiveness of glioma. The fraction of M2 macrophages was inferred using CIBERSORT. Correlation analysis found that the IFNGR score was positively correlated with the fraction of M2 (Figure 5A), in line with the results of transcription factor enrichment analysis. Besides, we found that the IFNGR score-high group had increased expression of immune checkpoints, including PD-L1, PD-L2, TIM3, and CTLA-4 (Figure 5B), consistent with the finding thatM2 macrophages express substantial immune checkpoint (36). Then, sample responsiveness to ICB therapy was evaluated. We employed TIDE and Submap algorithm to predict the responsiveness of samples to ICB and compared the results of the IFNGR-based groups with previously validated IFNG-related gene signatures-based groups. Based on the TCGA cohort, the IFNGR score-high group tended to respond to anti-PD-1 therapy (Bonferroni corrected *p* = 0.016), similar to the previously established IFNG-related gene signatures which have been validated by experiments (Figure 5C). Similar results were yielded using the CGGA325 cohort, with the IFNGR score-high group being more likely to respond to the anti-PD1 therapy (Bonferroni corrected *p* = 0.001) (Figure 5D). Therefore, these results indicated that an increased IFNGR score was associated with sample sensitivity to ICB treatment.

## Discussion

Gliomas include LGG and GBM, and their accurate diagnosis and effective treatment remain a challenge. In this study, we have developed a concise IFNG-related gene signature to characterize the prognosis of gliomas based on the expression of IFNGR1 and IFNGR2. As expected, the IFNGR score well represents the IFNG-related biological processes and extensively correlates with clinicopathological parameters that indicate a poor prognosis of glioma. Notably, we found that the IFNGR score was a robust prognostic biomarker of glioma (as well as high-grade glioma), and had the potential to screen ICB responders. Together, our work provides valuable information for the diagnosis, prognosis, and classification of gliomas and may help to optimize immunotherapy.

To our knowledge, we have for the first time explored the role of IFNGR1 and IFNGR2 in gliomas. IFNGR1 and IFNGR2 comprise the heterodimeric receptor for IFNG and their dysfunction is involved in various immune-related pathologies (7). One clinical study containing 213 patients and 733 controls has shown a correlation between IFNGR1−56C/T polymorphism and early onset of gastric carcinoma (11). A possible explanation is that individuals carrying the IFNGR1−56^*^T allele produce more IFNGR1, which renders cells more sensitive to IFNG, resulting in a more pro-inflammatory microenvironment upon H pylori infection. Besides, loss of tumor-suppressive transcription factor Elf5 promotes the growth and metastasis of triple-negative breast cancer through stabilizing IFNGR1 (14). On the other hand, the IFNGR2 is involved in the regulation of Th1 and Th17 homeostasis, and the lack of which is associated with mycobacterial disease (12, 13). In line with these findings, we found that the IFNGR score was positively correlated with the malignant biomarkers of gliomas in terms of histology, WHO grade, and transcriptome subtype. In addition, GO analysis corroborates the pro-inflammatory microenvironment of the IFNGR score-high group, suggesting that the upregulation of IFNGR1 and IFNGR2 was associated with enhanced inflammatory and immune response in gliomas. Nevertheless, an active immune response does not necessarily benefit glioma patients. For instance, the immune cytolytic activity measuring the function of CD8+ T cell, and the IFNG response genes indicating activation of adaptive immune responses were negatively correlated with the overall survival of glioma patients (4, 37), which is in line with our findings. Therefore, manipulation of the immune response for long-term control of glioma growth requires a deeper understanding of the composition of the immune response and the specific tumor microenvironment of gliomas.

IFNG is a double-edged sword immune-modulator and its role in glioma remains controversial. On the one hand, IFNG involves in the differentiation of Th1 cells, maintains the Th1-type immune response, as well as enhances the cytotoxicity of T lymphocytes, making the IFNG gene signatures effective biomarkers of an activated anti-tumor immune response (9, 38–40). Nevertheless, studies revealed that IFNG promotes tumor immune evasion by upregulating PD-L1 in a JAK-STAT pathway-dependent manner, implying that tumors characterized by increased IFNG response may be sensitive to immune checkpoint blockade therapy (4, 8, 9, 41). Given that IFNGR1 and IFNGR2 are receptors indispensable in the IFNG-mediated activation of the JAK and STAT families (7, 10), it was plausible that the 2-gene signature characterizes the upregulation of the IFNG signaling pathway well, and that patients in the IFNGR score-high group were potential responders to ICB therapy. However, multiple mechanisms are involved in the formation of glioma immunosuppressive microenvironment. For example, TGF-beta is involved in the inhibition of antigen presentation, the function of antigen-presenting cells, and the activation of T cells (42), COX-2 and PGE2 participate in tumor growth and angiogenesis (43), as well as CCL2, recruits immunosuppressive cells such as regulatory T cells and MDSC (44, 45). Therefore, it is necessary to take into account the role of other immunosuppressive mechanisms while applying ICB in the treatment of gliomas.

Another interesting issue is about the polarization of macrophages in the glioma microenvironment. Tremendous studies have demonstrated that macrophages tend to acquire pro-inflammatory M1 phenotype when stimulated by IFNG (46, 47), and LPS, TNF, and IFN-β also drive macrophage polarizing to a pro-inflammatory phenotype (46). Instead, IL-4, IL-13, and glucocorticoid induce alternative activation of macrophages (46, 48). Although various IFNG-related inflammatory responses may be active in the glioma microenvironment with elevated IFNGR scores, macrophages with increased expression of IFNGR1 and IFNGR2 were preferentially subject to transcriptional regulation by NF-κB and STAT3. Wenjing Xuan et al. summarized that NF-κB and STAT3 skewing microglia in the glioma microenvironment to an alternative activation phenotype (49), indicating that macrophages with increased IFNGR1 and IFNGR2 were probably pro-tumoral M2. Recently, we have reported that in the glioma microenvironment, levels of IL4, IL13, IL10, and TGFβ are increased by the IFNG response, which constitute a regulatory network of inflammatory responses and ultimately drives macrophages toward M2-type polarization (50). Therefore, we presumed that more effort is needed to clarify the association between the type of inflammation in the glioma microenvironment and the functional state of macrophages.

## Conclusions

In conclusion, we have constructed a clinical valuable IFNG-related gene signature for gliomas based on large-size and multi-cohort samples. These findings were based on general bioinformatics analysis and reliable statistical methodologies, but further basic and clinical studies are needed to verify their validity as well as molecular mechanisms.

## Data Availability Statement

The datasets presented in this study can be found in online repositories. The names of the repository/repositories and accession number(s) can be found in the article/supplementary material.

## Author Contributions

YL and HJ conceived and designed the study. HJ provided analytical technical support and drafted the manuscript. YL participated in the production of charts and pictures and supervised the study. YL and XG revised the manuscript. All authors have read and approved the final manuscript.

## Conflict of Interest

The authors declare that the research was conducted in the absence of any commercial or financial relationships that could be construed as a potential conflict of interest.

## Publisher's Note

All claims expressed in this article are solely those of the authors and do not necessarily represent those of their affiliated organizations, or those of the publisher, the editors and the reviewers. Any product that may be evaluated in this article, or claim that may be made by its manufacturer, is not guaranteed or endorsed by the publisher.
